# “When a woman is pregnant, her grave is open”: health beliefs concerning dietary practices among pregnant Kalenjin women in rural Uasin Gishu County, Kenya

**DOI:** 10.1186/s41043-017-0130-0

**Published:** 2017-12-16

**Authors:** Roselyter Monchari Riang’a, Anne Kisaka Nangulu, Jacqueline E.W. Broerse

**Affiliations:** 1 Department of Sociology and Psychology, Moi University, School of Arts and Social Sciences, P.O. Box 3900-30100, Eldoret, Kenya; 2 Athena Institute, Faculty of Earth and Life Sciences, VU University Amsterdam, De Boelelaan 1085, 1081 HV Amsterdam, The Netherlands; 3 Commission for University Education, Red Hill Road, off Limuru Road, Gigiri, P.O. Box 54999 – 00200, Nairobi, Kenya

**Keywords:** Maternal nutrition, Pregnancy, Food beliefs, Health beliefs, Nutrition intervention, Child birth, Kalenjin, Uasin Gishu County, Kenya

## Abstract

**Background:**

Reducing malnutrition remains a major global challenge especially in low- and middle-income countries. Lack of knowledge on the motive of nutritional behaviour could ultimately cripple nutrition intervention outcomes. The purpose of this study was to investigate how health beliefs influence nutritional behaviour intention of the pregnant Kalenjin women of rural Uasin Gishu County in Kenya. The study findings provide useful information for culturally congruent nutrition counselling and intervention.

**Methods:**

In this qualitative study semi-structured interviews were conducted with 42 pregnant and post-natal (with children less than one year) Kalenjin women in selected rural public health facilities of Uasin Gishu County Kenya. Furthermore, key informant interviews took place with 6 traditional birth attendants who were also pregnancy herbalists, two community health workers and one nursing officer in charge of Maternal and Child Health (MCH) for triangulation and provision of in-depth information. Content analysis of interview transcripts followed a grounded theory (Protection Motivation Theory) approach, using software MAXQDA version 12.1.3.

**Results:**

Abstracted labour (big babies and lack of maternal strength), haemorrhage (low blood), or having other diseases and complications (evil or bad food) were the major perceived health threats that influence nutritional behaviour intention of the pregnant Kalenjin women in rural Uasin Gishu County in Kenya.

**Conclusion:**

The pregnancy nutritional behaviour and practices of the Kalenjin women in rural Uasin Gishu County act as an adaptive response to the perceived health threats, which seem to be within the agency of pregnant women. As a result, just giving antenatal nutritional counselling without addressing these key health assumptions that underlie a successful pregnancy outcome is unlikely to lead to changes in nutritional behaviour.

## Background

Maternal nutrition, especially during pregnancy, has a significant influence on the women’s health, pregnancy outcome and child survival. A malnourished pregnant woman has a greater risk of having a complicated delivery, of mortality due to post-partum haemorrhage, and of miscarriages or stillbirths, and is highly likely to deliver babies with intrauterine growth retardation (IUGR) or low birth weight (LBW) [1–3]. Babies born underweight, pre-term or with IUGR have increased risk of neo-natal deaths. Nearly half (45%) of all deaths among children under five are attributed to undernutrition, amounting to 3 million deaths each year [4].

Due to adverse outcomes of malnutrition, worldwide interventions aiming to reduce maternal malnutrition and its consequences have been implemented. In Kenya, the first Food Policy was developed in 1981 and it aimed at maintaining broad self-sufficiency in major foodstuffs and to ensure equitable distribution of food of nutritional value to all citizens [5]. Since then, the government has put in place several policy measures to address food security in the country including Kenya vision 2030, The Agriculture Sector Development Strategy (ASDS) 2010–2020, The National Food Security and Nutrition Policy (NFSNP) 2009, and the Kenya National Nutrition Action Plan (KNNAP) 2012–2017 [6]. One of the main objectives of the NFSNP is to increase the quantity and quality of food available, accessible and affordable to all Kenyans at all times and to achieve good nutrition for optimum health of all Kenyans, women and children included [7].

Despite the many policy interventions in Kenya, no significant change is observed in most nutrition indicators over the last 10 years. An estimated 2.8 million or one-third of Kenyan children under the age of 5 are stunted [8], and maternal deaths increased from 414/100,000 live births in the year 2003 to 488/100,000 live births in the year 2008. Micronutrient deficiencies are highly prevalent in Kenya. Children under five years are particularly affected by deficiencies in vitamin A (84% of children), iron (73.4%), and zinc (51%). Pregnant women are among the most vulnerable, with a high risk of iron deficiency (60% among pregnant woman) and vitamin A deficiency (39%) [7]. Nutritional deficiency in Kenya is higher in rural areas and poorer households [8], thus pregnant women and millions of children remain at risk. This clearly indicates that there is a gap between the nutritional advice and actual practice.

There are several challenges and factors that cripple the efforts of nutritional interventions. Maternal and child nutrition studies in Kenya have typically focused on socio-economic, demographic and health factors, such as severe illness and infections, having multiple and short interval births, maternal age and education, household food security and nutritional knowledge as determinants of nutritional status [9, 10].

However, it is indicated that the meaning of food is more than just a source of nutrition and it carries with it a range of symbolic meanings [11, 12]. Intentions of eating a particular food to specific individuals and cultural groups has a rationality (cause-effect reasoning) that is very strong, and without truly understanding this reasoning there is little chance of nutrition intervention success, especially if the nutritional advice is counter to the nutritional intention of the target group. Various studies that evaluate innovative practices and interventions have established that many of the innovative practices fail due to the lack of contextual embeddedness or cultural sensitiveness [13–15], i.e. when there is a cultural discordance between the community’s definition of food and bio-medical understanding of nutrition. Abubarkar et al. [16] recommend that interventions need not only to address direct causes, but should also take into account the context of the problem to make sure that an innovative practice would have the desired impact. It is against this background that this study was designed to understand the nutritional behaviour intention of the pregnant Kalenjin women of rural Uasin Gishu County in Kenya. The study specifically explores the health beliefs within the context of pregnancy food beliefs and practices. This information is critical to the development of dietary recommendations, nutrition programmes, and educational messages that will assist maternal health providers in constructing healthful diets and promote dietary change.

## Methods

### Description of the study area

Uasin Gishu County is one of the 47 counties of Kenya and is located in the western part of the country. It has 171 health facilities, of which 90 are public, and most of the facilities are concentrated within the county headquaters (Eldoret) [17]. The county is divided into six sub-counties and it covers a total area of 3345.2 km^2^ with a total estimated population of 1,023,656, comprising 50% male and 50% female [17]. The predominant settlement pattern is rural (64.1%). The Kalenjin are the predominant ethnic group with eight sub-tribal groups: Nandi, Kipsigis, Keiyo, Marakwet, Tugen, Pokot, Terik and Sebei [18]. The Nandi have the highest settlement, followed by the Keiyo.

The Kalenjin have a common staple diet: *Kimyet (ugali)* (a paste of cooked millet flour), native vegetables and *mursik* (sour milk mixed with charcoal dust, sometimes mixed with cow’s blood), supplemented with roast meat (usually beef or goat). Fish was also part of the traditional diet though largely limited to residents bordering the lake region community. The climatic conditions and soil type in this region are generally favourable for a wide range of livestock and crop production with an average rural land holding of 5 ha, hence it is commonly known as the country’s food basket [18]. However, the nutritional status of Uasin Gishu County is worse than the national norm. Some 11.5% of the Uasin Gishu population is underweight compared to the national level (11.0%) while 31.2% of children are stunted compared to 26.0% nationally [8] hence need to establish why the high prevalence rates of malnutrition despite the food-surplus in the county.

### Data collection

The study is part of a larger project in Uasin Gishu County on maternal and child nutrition and health. Data were collected between April and August 2015 in 23 rural and less cosmopolitan public health facilities selected from the six sub-counties. This aimed at realising cultural homogeneity and thus restricted the study from the facilities that border Eldoret Municipality, the neighbouring Trans-Nzoia and Kakamega counties (mainly habited by the Luhya community) and some parts of Ainabkoi sub-county which have a large population of the Kikuyu community. To diversify the responses (maximum variation within the Kalenjin population), the selected facilities are well distributed throughout the county. The method of data collection was qualitative: semi-structured interviews with pregnant and post-natal women and key informant interviews with various stakeholders.

### Semi-structured interviews

Semi-structured interviews were held with pregnant women and mothers of children of up to one year, who came for routine antenatal care (ANC) and Child Welfare Clinic (CWC) in the Maternal and Child Health (MCH) section in the 23 selected rural health facilities. The semi-structured interview guide gave room to probe and explore topics as they arose to gather insight and detail-rich information [19]. The participants were purposively selected. Eligibility criteria for the study participants depended on: pregnant or post-natal woman, a Kalenjin by birth, willing and able to participate, willing to be tape-recorded, able to give informed consent. Age diversity was also taken into account.

A total of 42 Kalenjin women comprising 14 antenatal clients and 28 post-natal clients with children less than one year old were interviewed. The demographic characteristics of the interviewees are shown in Table 1.

**Table 1:** Demographic characteristics of the respondents

Characteristic	Categories	Number of cases (*N* = 42)
Maternal status	Antenatal	14
	Post-natal	28
Parity	First pregnancy/child	13
	1–3	15
	4–6	11
	7–8	3
Age (years)	≤19	4
	20–24	16
	25–29	10
	30–34	7
	≥35	5
Religion	Christians	42
	Others	0
Marital status	Never married	8
	Currently married	30
	Separated	2
	Widowed	2
Educational level	Primary education	21
	Secondary education	16
	Post-Secondary education	5
Occupation	Student/pupil	4
	Business	7
	Farming	26
	Formal employment	1
	Others	4
Ethnic Affiliation	Nandi	31
	Keiyo	4
	Marakwet	5
	Terik	1

Most of the Kalenjin ethnic-groups were represented: the Nandi were the majority (*n =* 31), followed by the Marakwet (*n* = 5) and the Keiyo (*n* = 4). The Tugen and the Terik were represented by one respondent each. This is because the Nandi have the highest settlement in Uasin Gishu County, followed by the Keiyo and the Marakwets [18]. The age of the respondents ranged from 18 to 42, with the majority of the respondents (63%) between 20 and 29 years old. All respondents were Christians (the dominating religion in the area) with more than half (61%) working in the domestic informal sector as subsistence farmers. Most of them (88%) did not have beyond secondary education, most women were married (71%) and parity of the respondents ranged between 1 and 8 children.

The interview guide covered diverse topic areas including food restrictions during pregnancy, food recommendations during pregnancy, health beliefs associated with food recommendations and restrictions during pregnancy, respondents’ opinions on these food beliefs and major pregnancy nutritional advisers. All interviews were conducted by the first author in Swahili language, and they lasted for 25–60 min each. However, cases of technical terms were clarified by Community Health Workers (CHWs) who were natives. The data collection exercise was continued until saturation was reached [20] at a sample size of 42 responses.

### Key informant interviews

Key informant interviews (KII) were conducted to explore meanings and enrich the responses obtained from interviewees. Specifically key informants were asked about the following main issues: perceived restricted and recommended foods, in-depth information on underlying reasons and the food advice they give to pregnant women. In total, nine KII were conducted with Traditional Birth Attendants (TBA) who were also traditional pregnancy herbalists (*n* = 6), community health workers (*n* = 2), and a nursing officer in charge of MCH (*n* = 1). The nursing officer and CHWs were recruited from the busiest rural health facilities, whereas the TBAs/herbalists were recommended by interviewees through snowball sampling and were reached at their homes or market centres. The first author conducted all the KII. These interviews were conducted in English apart from those with TBAs/herbalists, which were conducted in mother tongue with the help of a pre-trained translator. Each interview took 85–100 min. All TBAs/herbalists accepted being tape-recorded, while the officer in charge of MCH and CHWs did not consent to tape recording: in this case extensive notes were taken.

### Ethical considerations

The investigator obtained informed consent from all respondents involved in this study. In the beginning, all aspects of the study were discussed with the Uasin Gishu County Director of health, county commissioner, and county director of education, and their approval was obtained. Informed consent (written) was obtained from all the participants of the study. The consent form explicitly outlines the aims and objectives of the study along with the strict confidentiality of the participants. Approval and research permit was also obtained from the National Commission for Science and Technology and Innovation (NACOST) Kenya.

### Data processing and analysis

The recorded audiotapes were transcribed. The transcripts and notes were coded and then “thematised” for patterns, pre-set themes, emerging themes and categories using MAXQDA software version 12.1.3 with each participant being identified with a pseudonym code. The themes were based on food that is recommended during pregnancy, food that is restricted during pregnancy, underlying health beliefs and respondents’ opinions on the beliefs. Protection Motivation Theory (PMT) as expounded by Boss et al. [21] was further used to analyse the food consumption behaviour of the pregnant Kalenjin women with regard to food beliefs.

Protection Motivation Theory (PMT) was originally developed in 1975 to explain the effects of fear appeals on health attitudes and behaviours [22]. Since then, PMT has been revised and successfully applied in different fields of study [23–26]. All these modified versions of PMT share the idea that motivation towards protection results from a perceived threat and the desire to avoid the potential negative outcome. The theories also share a cost-benefit analysis component in which the individual weighs the costs of taking the action against the expected benefits of taking that action. This is to say, increases in the perceived level of fear consistently result in increases in acceptance of the proposed adaptive behaviour or intention. Also, increments in perceived response efficacy increase the intentions to select the adaptive response. Thus, the protection motivation concept involves any threat for which there is an effective recommended response that can be carried out by the individual.

**Fig. 1:**
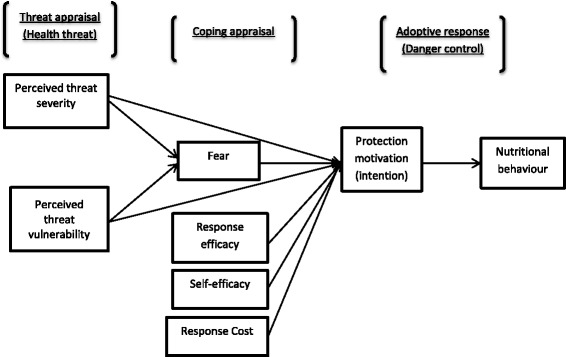
Modified Protection Motivation Theory (MPMT)

For this study, a Modified PMT (MPMT) model (Fig. 1) was developed based on core and full nomologies of PMT as proposed by Boss et al. [21]. The MPMT uses two constructs of PMT: threat appraisal and coping appraisal. Threat appraisal includes severity of health threats (perceived severity) and the perceived probability that health threats will occur (perceived vulnerability). Coping appraisal includes the perceived ability of a coping behaviour (e.g. consuming or restricting certain foods) to remove the health threat (response-efficacy) and the individual’s perceived ability to carry out the coping behaviour (self-efficacy, e.g. actual consumption or restriction of certain foods). However, to decide to adopt the recommended coping response (positive self-efficacy), one must believe that performing the coping response will avoid the danger and that one has the ability and will to perform the response. These considerations must outweigh the costs (e.g. accessibility and affordability of recommended food) of performing the coping response. On the other hand, a fear appeal does not just increase threat but would also increase efficacy by giving a respondent a path to address the threat. Thus the outcome of the threat and coping appraisal is the intention to initiate, continue, or inhibit the applicable adaptive responses. Intention in turn predicts nutritional behaviour.

The perceived health threats established in this study were pregnancy-related complications, that is, abstracted’ labour, haemorrhage, disease and complications of the mother and/or the baby as explained in the results section. However, the MPMT model eliminates the maladaptive rewards construct of Boss et al.’s [21] full model, because it does not fit in the result findings.

## Results

In this section, an application of PMT to the prediction of nutrition intentions and behaviour basing on food believes is described. First, food restrictions and recommendations during pregnancy (copying appraisal) were analysed and categorised according to the underlying health consequences (health threat appraisal) as well as perceived severity and perceived vulnerability. This is followed by an analysis of the respondents’ opinion and influence of these beliefs (efficacy) on their nutritional intention and behaviour.

### Perceived health threat, vulnerability and severity appraisals

Three major perceived health threats were established in this study: abstracted labour, haemorrhage, and disease/complications of mother and/or baby. Below we discuss the perceived severity in terms of the consequences of the health threats as perceived by the participants. In all instances the respondents indicated that they were highly vulnerable to these threats, in other words they considered themselves very susceptible to the mentioned health threats.

#### Perceived health threat: Abstracted labour

In this study, abstracted labour was considered the most important health threat, and two reasons for this were given: (1) having a big foetus, and (2) mother lacking strength or having a weak body. The danger of abstracted labour is that it may lead to a Caesarean Section (CS), episiotomy (big tears on the birth canal, in the case of a big foetus), placenta retention (in the case of lacking strength), stillbirth, maternal morbidity and mortality. Furthermore, birth attendant midwives were said to harass women in case of abstracted labour.

*“And if you are not able to push during birth you will be beaten thoroughly*.*”* (150510_002)

Many respondents particularly referred to a CS as a daunting consequence.

*“What if the baby overgrows inside there? … You will obviously go to theatre!”* (150621_001-53)

“*If you do not feed well on the pregnancy recommended food, you will not have strength for pushing out the baby during birth. They will demand an operation so that they remove the baby.”* (150602_001-27)

Several reasons why the women feared CS emerged. The greatest fear expressed by these women is that CS is a risky process, which may even lead to death of the mother or the child, and if the mother dies, the child will be left motherless.

*“You can either die, or survive, and if you die you will leave your baby suffering. You know with CS survival chances are fifty-fifty.”* (170621_002-30)

Many respondents also felt that if a woman undergoes CS, the scar will take a long time to heal and this will restrict her diet and normal daily duties.

*“If a mother is operated, she cannot perform heavy routine duties as required by the Kalenjin culture, she cannot even resume her normal diet; the stiches might tear off and demand re-stitching.”* (150615_002-15)

#### Perceived health threat: Haemorrhage

Haemorrhage was another major perceived health threat found in the study. The most important reason for haemorrhage believed by these respondents is *having less blood* during the pregnancy period. Haemorrhage during birth may lead to maternal death, lack of strength to push out the baby, poor haemoglobin results, referral cases and blood transfusion as narrated below.

*“When your blood is less, it will only help you to get a baby, thereafter, you will bleed until your body dries up and you die”.* (150602_001)

Fear of haemorrhage is also accelerated by the health practitioners at the MCH clinic. As mentioned by a key informant at the MCH, it is a requirement for every pregnant woman to undertake a haemoglobin (Hb) test during the first and last ANC visit, and if the Hb level is too low, appropriate nutritional therapy is prescribed to the client. The client will come back for a review/check-up after 4 weeks to confirm whether the Hb has improved. These consistent Hb checks seem to create anxiety and fear of having “less blood” among the pregnant women.

*“I do not even understand why, because blood is the most sensitive thing in the body of a pregnant woman. Every time we come for clinic they always ask to check blood levels. If it is less and the delivery date is not close, they list for you the foods to eat. So as per my opinion, a pregnant woman should have a lot of blood and it should not reduce at all.”* (150609_002-80)

The challenges facing health facilities, especially in the rural areas, seem to accelerate this fear of less blood. An interview with a nursing officer in charge of MCH reported that the primary healthcare facilities (health centres and dispensaries), which are commonly found in rural areas, are not equipped with theatre and blood transfusion kits. As a result, they do not take the risk of conducting a delivery if a woman’s Hb level is 8 grams per deciliter or less, but refer the cases to a bigger facility. There is only one public facility in Uasin Gishu County (Moi Teaching and Referral Hospital (MTRH)), therefore a woman will be better positioned to deliver at a local facility (which is nearer) if her Hb is above 8 grams per deciliter. It was established that referral cases and the possibility of a CS and blood transfusion increases fear and anxiety in a pregnant woman.

“*During the 7th month pregnancy of my other girl that this child follows, my blood was four points (Hb4). So they told me that I will go to theatre. … Fortunately, by the time I was due, my blood had risen to 8 points. But they had to refer me to MTRH when I was due because my blood was less. It was so fortunate that I didn’t go to theatre. God is real good.”* (160615_05-74)

At the same time, a TBA reported that they were told in a seminar not to attempt a delivery when they notice that the Hb is recorded 8 or below on a woman’s antenatal card but they should refer. However some circumstances force them to attempt it and probably refer when their efforts fail as explained:

*“If you see a woman looking weak, we refer her for medical check-up. I even know how to read her medical card, we were told if her blood is less than 8 we should not attempt that delivery, although others come at night when it is raining and there is no transport and they beg for your help. We have no choice but to conduct the delivery.”* (150412-003-45)

#### Perceived heath threat: Disease and complications of the mother and/or the baby

Another health threat reported by respondents is disease and complications of the mother and/or her baby. The main reasons for this were eating “evil food” or “bad food”*.* Eating such food was believed to be a severe threat because it can lead to maternal or child death, misfortunes, complicated pregnancy or delivery and maternal or child sickness. For example, some slaughter circumstances of an animal were associated with “evil spirits” that can be transferred to a pregnant woman and cause bad omen. The slaughter causal circumstances established during the study include an animal slaughtered due to pregnancy-related complications such as an abortion, a stillbirth, placenta retention, or death during birth. If such meat is eaten during pregnancy, the woman will encounter similar pregnancy complications.

*“If a cow was giving birth and in the process it dies, a pregnant woman cannot eat its meat. Or if a cow is pregnant and a calf dies in the uterus or the placenta refused to come out its meat cannot be eaten by a pregnant woman. They say that that “bad blood” of the cow will follow her and she will be like that cow during birth.”* (150602_001-80)

Other slaughter circumstances associated with evil spirits include: an animal that strangled itself by a rope and died, its meat will make the umbilical cord coil around the baby’s neck and cause a stillbirth; a sick or dead animal from unknown reasons is contagious and might transmit its disease to the mother or child; an animal with any deformity will make a woman give birth to a child with similar deformity; and a stolen animal will make a woman give birth to a thief child.

“*You cannot know what killed that animal, say like if it died of a disease and you eat it? You can also die from that disease.”* (150617_006-13)

Other foods are not associated with evil spirits, but are believed to be “bad” because they cause health complications to either the mother or the child in various other ways. Such foods include sheep meat, sheep’s head, fatty meat, oily food, sugary food and salty food. Sheep meat is believed to make the baby develop allergies such as rashes after birth whereas the sheep’s head is believed to cause a blocked nasal passage of the child, making the child breath with difficulty like a snoring sheep. On the other hand, sugary food, oily food or soil/soft stones if consumed by a pregnant woman will make the newborn develop colic. To other respondents, if a woman eats sweet food such as honey, sugarcane, sweets, ripe bananas and sugared beverages during pregnancy, her child will be oozing a lot of messy saliva on her/his clothes.

*“I did not eat a sheep meat, even ‘gogo’ (mother-in-law) told me not to. You know if you eat sheep, when you give birth, the meat brings rashes on your baby’s skin, like those of an allergy.”* (130707_005-106)

*“They cause ‘surunda’ – this stomach that disturbs newborns (colic pain). Ripe bananas are not good to eat when pregnant.”* (130708_001-118)

Some respondents believed that consumption of too oily food and eggs causes blood pressure problems in the pregnant woman and even the child. Too much oil and sugar are also believed to cause malaria.

*“A lot of oil is not good, the day of birth you will have a malaria attack because you have eaten a lot of oil until your stomach is full of oil and this brings malaria. If you are caught by malaria you might die.”* (150617_006- 13)

Vegetables that were grown on burned soil are forbidden to pregnant women. It is believed that she will give birth to a baby with burn-like rashes on the skin. Such rashes are very dangerous: a child might die because they are not bio-medically treatable but can be treated with herbs. Taking salt or soda ash during pregnancy will cause the baby to have rough, dry and cracking skin which will eventually start to peel off like that of a snake. Consumption of alcohol and cigarettes during pregnancy were believed to cause low birth weight because they suck the baby’s blood, affect the baby’s brain, and cause a stunted and retarded child.

### Perceived response efficacy and self-efficacy

Various perceived response efficacies and self-efficacy of coping with the above-mentioned health threats were established in the study as described in this section.

#### Perceived coping appraisal to abstracted labour

As stated earlier, abstracted labour is believed to be due to a big foetus and/or lack of strength of the mother. Thus the perceived response efficacy of coping with abstracted labour as believed by these respondents is first of all to maintain a small foetus in the uterus. This can be achieved by restricting consumption of protein and energy rich food during pregnancy, including eggs, meat, avocadoes, fatty food, cooked bananas and *moboriet.* 1

*“You should not eat strong strong things like eggs when pregnant. They make the baby grow big and then you will not be able to give birth.”* (R130709_001)

It was also established that TBAs/herbalists prescribe herbs to regularise the size of the baby especially if it has been established as being too big.

*“If you are pregnant, you are supposed to visit a TBA at least monthly. The baby might be dispositioned or oversize and you will not know. These women (TBAs) when they touch your stomach they can notice. They will reposition the baby and prescribe herbs to reduce the baby if too big.”* (150602_001-80)

Lack of strength at birth is perceived to result from a weak maternal body. The perceived response efficacy to maintain a strong body during pregnancy is by feeding well on the pregnancy recommended foods especially those rich in energy. For example *Ugali,* 2 especially the one made from sorghum and finger millet flour, is highly recommended during pregnancy because it is believed to be “stronger” than the usual *ugali* made from maize flour:

*“I use porridge a lot, it makes you have energy. If you don’t eat well when you are pregnant, you become weak until that day of delivery you will not be able to push out the baby.”* (150615_002_98)

Other items believed to give strength to a pregnant woman include porridge, potatoes, rice, boiled maize and herbs. Food believed to supply less energy such as rice and potatoes should only be eaten once in a while probably because of the “strong pregnancy urge” to eat them. Traditional vegetables and milk are believed to add strength to the woman because “they build body”. It was also stated that having enough blood makes a woman strong during pregnancy and at birth.

### Perceived coping appraisals to haemorrhage

The main cause of haemorrhage according to many respondents is having less blood. Thus the perceived response efficacy of coping with haemorrhage believed by the respondents is to maintain high blood during pregnancy. The consumption of indigenous vegetables, mostly leafy and green in colour, including pigweed, black nightshade, spider plant, spinach, white vine spinach, pumpkin leaves, and cowpea leaves, during pregnancy is highly recommended not only by the so called *gogos* (grandmothers) but has also been adopted by the health practitioners as nutritional therapy for pregnant women with lower Hb levels. Other items believed to increase blood, as mentioned by various respondents, include liver, fruits, animal blood, milk, red beans, porridge prepared by flour of finger millet mixed with sorghum, iron supplements (issued at the ANC clinic), herbs and red soil/stones.

*“Traditional vegetables, spinach and liver add blood, even when you come to clinic, you are told to eat them. If you do not eat these vegetables in plenty, your blood will reduce.”* (150510_002)

Milk mixed with blood is also believed to raise blood levels very fast. As a result it is highly recommended to pregnant mothers whose Hb is discovered to be below the required levels. Alternatively animal blood can be cooked and fed to a pregnant woman when her Hb is low.

*“When you are pregnant and you are told your blood is less, you need to go to the butchery, you collect blood of the cow, sheep or goat depending on what animal they slaughtered, you ferment it with fresh milk, you stock it and you be drinking it daily. It helps your blood to increase fast.”* (130707_002-104)

To some respondents, eating red soil and red stones during pregnancy increases blood and others believed that soil sucks mother’s blood resulting in anaemia.

“*I was having four pints of blood when I was pregnant of my other daughter … And that is why I was telling you that soil finishes blood. Because I used to eat a lot of it even before I conceived that pregnancy.”* (160615_05-74)

#### Perceived coping appraisal to disease and complications of mother and/or baby

Many diseases and complications of mother and/or child were believed to be caused by eating “evil food” or “bad food”. Therefore the established response efficacy of coping is to avoid eating such food, conducting cleansing rituals, and using herbs to protect, neutralise or treat the effect. For instance, women when pregnant were advised to be cautious about eating any meat.

On the other hand, traditional herbs were highly recommended to a pregnant woman, whether sick or not. They are believed to prevent the newborn from getting sick and to prevent the transfer of disease from mother to foetus. The herbs also clean the baby’s skin and stomach while in the uterus so that it will be born healthy, with a clean smooth skin.

*“If you don’t consume traditional herbs when you are pregnant, your child will not be born with good health. The baby will be susceptible to seasonal diseases. If it is malaria or cough season she/he cannot escape. Your baby will also not be bothered with stomach ache (colic pain) or skin rashes if you were using these herbs during your pregnancy.”* (150610_003-77)

### Perceived response costs

Despite the fact that all respondents were in support of the pregnancy food prescriptions, some constraints appear to make it difficult to comply. The greatest constraint reported by these women was the gastro-intestinal discomforts that caused feelings of vomiting, nausea and acidic stomach and bad smell. The reported food that is recommended yet believed to cause this problem include fermented milk and beans.

*“You can be advised many things but the body refuses, so you just eat what your body wants to.”* (130709_006-129)

Other respondents reported climatic constraints. During the dry seasons (January–March), some food crops, especially the green native vegetables, are scarce, and if available they are expensive in the market. Pregnant women during this season tend to suffer shortage of greens. Though some respondents felt some few homesteads are poor and cannot afford to access all the recommended food, overall however the majority of pregnant women interviewed claimed that food accessibility is not a problem.

Most respondents reported that it is safe for a pregnant woman to avoid eating restricted food, but some were compelled to eat them due to certain reasons. The established reasons were hospital recommendations or strong pregnancy urge. However they were eaten sparingly.

*“I find it a wise idea not to eat eggs when pregnant. You should eat maybe once in a while … say after a month or two months if you have that strong urge, otherwise, you are supposed to control yourself because the baby will grow excessively big.”* (130707_004-105)

*“Like now, the pregnancy of this child was pushing me to eat eggs, so I could eat when nobody is watching me. Otherwise if you are caught you will be quarrelled.”* (150510_002)

“*Yes I ate eggs because I was told to eat them at the hospital, but I mixed with in ‘chapati’ or kales. But I ate twice for the entire pregnancy.”* (130709-002-126)

## Discussion, conclusion and recommendations

This study examined the perceived health threats that enhance nutritional intention and behaviour in regards to food beliefs during pregnancy among the rural Kalenjin women of Uasin Gishu County Kenya. The findings clearly confirmed the Kalenjin saying *“ngotomono chebioso ko yatat kererit nyi”,* meaning, *“When a woman is pregnant, her grave is open”*, i.e. pregnancy and childbirth is considered to be a health threat in the sense that the pregnancy may end up with the death of the foetus, the woman and/or her newborn baby. The pregnancy food beliefs and practices are an adaptive response to these health threats, which seem to be within the agency of pregnant women. The fear of death during birth is actually not unrealistic in the Kenyan situation since the maternal mortality rate is very high at 488 maternal deaths per 100,000 live births [27] and 56% of infant deaths in Kenya occur during the first month of life [8].

From the critical evaluation of the various perceived health threats that emerged from the results, fear of having a complicated labour that may lead to a CS, maternal death, or stillbirth was the key fear that drives the Kalenjin women to subscribe to dietary precautions and beliefs. The research findings reveal that poor health outcomes can arise due to abstracted labour (big babies and lack of maternal strength), haemorrhage (low blood), or having other diseases and complications (evil or bad food), which can be avoided by observing diet during pregnancy. However, from the critical evaluation of literature, various factors can be used to justify these fears held by the Kalenjin women as enumerated below.

This study was carried out in a rural area in Kenya that is faced with institutional and infrastructural challenges. The biggest institution in the rural areas (Health Centre) for instance only provides primary healthcare and basic first aid for obstetric complications. They are not equipped to conduct complicated deliveries such as obstructed labour or surgery. They are headed by a Clinical Officer who is not qualified to conduct surgery in case of an emergency [28]. It has also been established that the referral systems in case of an emergency are very poor and this is exacerbated by poor rural road access [28–30]. Besides, most deliveries in Uasin Gishu County (48%–66%) are conducted at home by either the TBAs, neighbours or the mother herself [8] which might be problematic in a case of a complicated or abstracted labour. If operative delivery to ensure a healthy birth is not available to women who need it, both mother and baby are at risk, and even if operative delivery is accessible, affordable and safe, anaesthesia and laparotomy increase the risk of maternal morbidity [31]. As a result, fears of a complicated delivery by these respondents are justified. However, the extent to which the Kalenjin women experience obstetric complications and the extent to which eating certain foods contribute to abstracted labour need to be established. In literature, one of the factors associated with obstetric complications is Female Genital Mutilation (FGM). Researchers have established that the scar tissue left after FGM may contribute to obstructed labour, since fibrous vulva tissue fails to dilate during contractions [32]. The prevalence rate of the FGM among the Kalenjin community is reportedly high at 40.4% [27]. Furthermore, haemorrhage (a leading cause of maternal death in developing countries) may result from tearing through scar tissue that is left as a result of FGM. FGM is a social custom and value by these women may not be perceived as pathological, making them believe that obstructed labour is caused by different reasons, such as big babies, less blood or lack of strength which is caused by eating or not eating certain foods. Fear of a complicated labour and delivery was also established in Ghana, Ethiopia and Nigeria [33–35].

However, the Kalenjin believe that a complicated delivery can, among others, be prevented by *avoiding big babies*, and big babies can be avoided by restricting consumption of eggs, meat, milk, *moboriet* and cooked bananas, among other foods, during pregnancy. In addition, TBAs/herbalists prescribe herbs that aim at reducing the size of the baby if it is established that it is too big, to enhance easier delivery. Most of the restricted foods are protein and energy rich. Scientifically, if such foods are consumed more than it is used, the extra is converted and stored as fat in the body and gains weight that can lead to obesity [36]. Hence, restricting pregnant women from consuming such food in excess is one way to prevent an overweight baby. However, it can be questioned whether eating eggs and other nutrient-rich foods will always lead to big babies and to what extent maintaining a small foetus is an effective means of surviving pregnancy and delivery. In reality, only excessively heavy babies of more than 4–4.5 kg are a reason for undergoing a caesarean section [37] which is quite a rare condition. In Kenya, only 3.6% of the babies have a birth weight over 4 kg [38]. Furthermore, babies with a low birth weight are also an indication for a CS [39], which means that attempts to keep the baby small to avoid a caesarean section might actually work out to the contrary. Further, the commonly established risk factors significantly associated with CS deliveries in Kenya include short maternal structure, women from households of high socio-economic status, age of the mother and lack of utilisation of appropriate maternal health care services [30, 40]. Nevertheless, with limited knowledge, it appears to be a very rational decision for pregnant women to prevent themselves from dying during childbirth by preventing their babies from growing big. Fear of big babies resulting in possible death of the mother during birth was also established in Ethiopia, Nigeria, Zambia, Kenya and Ghana [12, 33–35]. However, the foods believed to make the baby big vary.

The results of this study also express the importance of *having enough strength* (strong body) for a pregnant woman especially during birth, as a means of preventing a complicated labour. Most of the foods believed to provide strength (*ugali*, porridge and potatoes) is indeed energy-giving. Milk and traditional vegetables, which are also associated with energy-giving, actually build the body and increase blood, which is vital in energy production. To these women, energy is vital during pregnancy. A weak woman will be fatigued and will not be able to push out the baby during birth. This is true because when there is an increase in the weight of a woman, the body mass ratio and body metabolic reactions also increase, thus requiring more energy. It has been established that about 27% of the 12.5 kg total pregnancy weight gain is stored as maternal fat, presumably to provide an energy reserve during the last part of pregnancy when foetal growth requirements are highest and during birth when more energy is vital [41]. Furthermore, the risk of spontaneous preterm birth and underweight births increases in women with a low rate of weight gain [42]. The fact that most deliveries (48%–66%) in Uasin Gishu County are conducted at home by the TBAs, neighbour or the mother herself [8] means that if a mother’s strength lowers during birth, the TBAs cannot help because they do not have glucose drips to boost their energy. However, the possibility is that maternal energy intake indirectly reflects other diet characteristics, such as nutrient density and dietary diversity [43, 44] merits more attention. None of the interviewed women mentioned that repeated pregnancy, combined with heavy work, also make a woman weak and sick [36].

To maintain *enough blood* (iron) during pregnancy according to these women is another factor believed to prevent a complicated labour and delivery that might lead to death. This belief is close to the current scientific insights: iron and folic-acid deficiency anaemia is very common in pregnancy and is associated with maternal death. A study by Ties Boerma and Mati [3] in the coastal region of Kenya established that iron deficiency was among the leading causes of maternal deaths. Post-partum haemorrhage (PPH) on the other hand is the single leading cause of maternal mortality and morbidity in Africa and other developing countries. More than half of all maternal deaths occur within 24 h of delivery, mostly from excessive bleeding [45]. Furthermore, rural health facilities, which are easily accessible, are not equipped with blood transfusion kits in case of an emergency blood transfusion incident [28]. From literature, it is known that people with good iron stores recover rapidly from losses of blood and iron stores are replenished when people eat iron-containing foods [36]. The native vegetables, spinach, liver, animal’s blood, red beans, fish and finger millet porridge which are believed by these women to increase the amount of blood are actually rich iron-containing foods, which is a micronutrient that contributes vitally to adequate haemoglobin levels [36]. This prevents the pregnant mother from having iron deficiency anaemia and its consequences for herself and her baby. Therefore, the cultural practice of the Kalenjin women to eat these foods during pregnancy should be enhanced by pregnancy nutrition interventions. However, there is a need to investigate whether the style and cooking habits of the Kalenjin women actually retain the essential nutritional components of these vegetables and whether it is safe to drink uncooked animal blood (mixed in fresh milk). Iron deficiency anaemia is caused not only by lack of iron in the diet, but can also be made worse by blood loss from hookworms, excess menstruation, abortion post-partum haemorrhage and injuries [36]. The interviewed women did not seem to be aware of this. On the other hand, a child born to an anaemic mother may also have a very low reserve of body iron, which can lead to low birthweight, and is likely to develop anaemia in the first few weeks of life which can lead to death [36]. However, the respondents are not convinced that there is a there is a relationship between anaemia and low birth weight, or risks other than haemorrhage. It is therefore necessary to create an understanding of the relationship between anaemia and foetal development to this community. A belief in the importance of iron was also established among pregnant women in Ghana, and traditional green vegetables were believed to provide blood or prevent anaemia [46].

Other food restrictions and recommendations established in the results aim at *protecting the mother and the child from getting sick*, and the food established to cause sickness include too much sugar, salt, oil, sheep meat and sheep’s head. Sheep meat is a fatty meat that is associated with a lot of oil. For as much as the health reasons associated (causing colic pain, dry crackly skin, oozing saliva, allergic rashes and nasal blockage to the baby) might not be valid, research indicates that consumption of foods high in saturated industrially produced trans fats, salt and sugar are responsible for 40% of all deaths associated with non-communicable diseases (NCDs) every year [47]. Hypertension disorders (one of the NCDs), for instance, is one of the commonly reported direct causes of maternal deaths in developing and developed countries [45]. Excess salt consumption can also lead to oedema, a common condition among pregnant women. However, it should be noted that salt and oil in moderation is essential for nerve and muscle function. Restricting consumption of excess fat, salt and sugar have also been established in other African countries including Burkina Faso and Ghana, but the beliefs associated with their consumption vary [33, 46, 48].

Herbs are also recommended here as immunity and Hb boosters to the mother, to protect against “evil eyes”, to prevent mother-to-child disease transfer and to enhance delivery of a healthy and smooth baby. The use of herbs during pregnancy is widely spread in Africa [12, 49–52]. However the question is: what are the compounds in these herbs and how safe are they for human consumption, especially during pregnancy when the human body is vulnerable?

Similarly, respondents also expressed fears of a complicated delivery as a result of consuming a sick or dead animal. Part of the reasoning expressed by these women is that the animal might have died from a contagious disease that could infect the foetus or the mother. When translated to the scientific perspective, this belief makes much sense: the cause of death of the animal might have been a fatal illness or infection that might make the mother sick.

Alcohol consumption and cigarette smoking during pregnancy is restricted among the Kalenjin women. Alcohol is believed to cause retarded children or underweight birth, or one can give birth to a drunkard child. This belief is not far from reality. Alcohol freely crosses the placental barrier and it causes foetal alcohol spectrum disorder (FASD) and can damage the developing baby’s cells resulting in lifelong physical and mental disabilities [53]. However, they are informed that there are no safe limits for the use of alcohol during the pregnancy period Similarly, pregnant women who are exposed to tobacco smoking have a higher risk of spontaneous abortions, stillbirths and ectopic pregnancies than non-smoking women [54], although the respondents seem not to be informed about this.

In this study we interviewed mothers who had delivered within the past 12 months, pregnant women, TBAs, CHWs, and nursing officers in charge of MCH. A limitation of the study, however, is that the results of the qualitative data based on a small purposively selected sample may not be representative of the entire population of Kalenjin pregnant women in Uasin Gishu County. Despite these limitations, it is remarkable to note that this study provides important insights on perceived health threats that drive nutrition behaviour intention among pregnant women within rural Uasin Gishu County in Kenya. Moreover, the use of qualitative techniques allowed us to gain a better understanding of health beliefs on pregnancy and delivery, and was used to triangulate findings generated by the semi-structured interviews.

## Abbreviations

ANC: Antenatal Clinic
ASDS: Agriculture Sector Development Strategy
CHWs: Community Health Workers
*CS*: Caesarean Section
CWC: Child Welfare Clinic
FASD: Foetal Alcohol Spectrum Disorder
FGM: Female Genital Mutilation
HB: Haemoglobin
IUGR: Intra Uterine Growth Restrictions
KII: Key Informant Interviews
KNNAP: Kenya National Nutrition Action Plan
LBW: Low Birth Weight
MCH: Maternal and Child Healthcare
MPMT: Modified Protection Motivation Theory
MTRH: Moi Teaching and Referral Hospital
NACOSTI: National Commission for Science, Technology and Innovation
NCDs: Non-Communicable Diseases
NFSNP: National Food Security and Nutrition Policy
PMT: Protection Motivation Theory
PPH: Post-Partum Haemorrhage
SEKU: South Eastern Kenya University
TBAs: Traditional Birth Attendants
VU: Vrije Universiteit Amsterdam

## Footnotes

1 Cold *ugali*, usually leftovers from the previous day, normally taken the next day for breakfast.

2
*Ugali* is the local staple food that is most often made from maize flour or a mix of maize flour with other types of flour. It is cooked with water until a paste or dough-like substance is attained.
